# A Comparative Analysis of Major Cell Wall Components and Associated Gene Expression in Autotetraploid and Its Donor Diploid Rice (*Oryza sativa* L.) under Blast and Salt Stress Conditions

**DOI:** 10.3390/plants12233976

**Published:** 2023-11-26

**Authors:** Zitian Leng, Keyan Liu, Chenxi Wang, Fan Qi, Chunying Zhang, Dayong Li, Ningning Wang, Jian Ma

**Affiliations:** 1 Faculty of Agronomy, Jilin Agricultural University, Changchun 130117, China; 18943776363@163.com (Z.L.); lky155662949882023@163.com (K.L.); 15947157832@163.com (C.W.); fan711998@163.com (F.Q.); zhangchunying@jlau.edu.cn (C.Z.); 2 Jilin Provincial Laboratory of Crop Germplasm Resources, Changchun 130117, China; 3 College of Plant Protection, Jilin Agricultural University, Changchun 130117, China; lidayong@jlau.edu.cn

**Keywords:** autotetraploid rice, cell wall major components, *Magnaporthe oryzae* stress, salt stress, gene expression

## Abstract

Whole-genome duplication is a significant evolutionary mechanism in plants, with polyploid plants often displaying larger organs and enhanced adaptability to unfavorable conditions compared to their diploid counterparts. The cell wall acts as a primary defense for plant cells against external stresses, playing an essential role in the plant’s resistance to various stressors. In this study, we utilized both autotetraploid and its donor diploid rice (*Oryza sativa* L.) to analyze their phenotypic differences comparatively, the composition of key cell wall components, and the expression of related genes under normal conditions, as well as under stress from *Magnaporthe oryzae* (*M. oryzae*) and salt. Our findings indicated that autotetraploid rice exhibits significantly larger phenotypic characteristics under normal conditions than diploid rice. At the seedling stage, the lignin, cellulose, hemicellulose, and pectin levels in autotetraploid rice were markedly lower than in diploid rice. Additionally, 24 genes associated with major cell wall components showed differential expression between diploid and tetraploid rice. At the filling stage, the lignin and pectin content in autotetraploid rice were significantly higher than in diploid rice, while the levels of cellulose and hemicellulose were notably lower. Under *M. oryzae* stress or salt stress, autotetraploid rice showed smaller lesion areas and less wilting than diploid rice. The increased lignin content in autotetraploid rice under *M. oryzae* stress suggested a stronger adaptive capacity to adverse conditions. Compared to salt stress, *M. oryzae* stress induced more differential expression of genes related to major cell wall components. In this study, we explored the differences in the major cell wall components of diploid and homologous tetraploid rice under various treatment conditions. This study provides valuable insights into understanding the cell wall’s adaptive mechanisms in autotetraploid rice when facing blast disease and salt stress, and it reveals the differential gene expression linked to these adaptive capabilities.

## 1. Introduction

Whole-genome duplication (WGD) is a distinctive mutational event leading to the complete duplication of an organism’s genetic material [1]. WGD is observed across various significant lineages of land plants and is particularly prevalent in angiosperms [2]. Based on the origin and extent of WGD, polyploids can be classified into two categories: heteropolyploids and homopolyploids. Heteropolyploids emerge from doubling chromosomes in hybrid offspring resulting from crosses between different species. Conversely, homopolyploids form through the duplicating of the entire genome within a single species [3]. Notably, self-pollinating crops such as rice (*Oryza sativa* L.) and wheat (*Triticum aestivum* L.) predominantly undergo polyploidization as homopolyploids. The occurrence of WGD profoundly influences plant phenotype, leading to significant alterations in their observable characteristics [4]. Polyploidization in plants leads to an increase in cell size, which confers advantages for cell surface-related activities, contributes to the development of larger plant organs, and alters the plants’ ability to adapt to adverse conditions [5]. It also induces structural and functional changes in the genome, including chromosomal rearrangements [6], gene loss [7], and epigenetic reprogramming [8], all of which have a significant impact on gene expression [9]. The genomic and transcriptomic changes in polyploid cells exhibit non-linear patterns associated with their adaptation to external stresses [10]. Overall, whole-genome duplication plays a crucial role in developing novel traits and increasing biological complexity in plants [11].

External environmental stress significantly affects the growth and development of plants, which mainly include biotic and abiotic stress. Biotic stress refers to the impact of living organisms, such as pests, diseases, and weeds, on plant health. These organisms can cause damage to plants by feeding on them, competing for resources, or transmitting diseases [12]. Abiotic stress refers to the impact of non-living factors, such as temperature, light, water, salt, and climate, on plant growth and development. These elements can influence plants’ physiological and biochemical processes, affecting their growth rate, flowering time, fruit maturity, and yield [13]. Several studies have demonstrated that both natural and artificial polyploids have the potential to enhance plant tolerance to biotic and abiotic stresses, positively affecting plant growth and yield [9]. In terms of salt stress, Wang et al. (2021) [14] observed that tetraploid rice exhibited better salt tolerance than its donor, diploid rice. This enhancement was attributed to tetraploid rice’s more rapid and robust induction of stress-responsive genes, including those associated with the jasmonic acid pathway. Furthermore, Mehlferber et al. (2022) [15] discovered that polyploid, Arabidopsis (*Arabidopsis thaliana*), exhibited greater resistance to the model pathogen, *Pseudomonas syringae* pv. Tomato DC3000, regardless of whether it was inoculated with microorganisms or not. In contrast, diploid Arabidopsis required microbial induction to trigger an adequate defense response. These findings suggest that whole-genome replication in polyploid plants enhances immune responses and safeguards them against pathogens invasions.

The cell wall is the primary defense barrier between the plant cell and its external environment. It is composed of various polysaccharides such as cellulose, hemicellulose, pectin, and aromatic compounds like lignin [16]. These components provide structural integrity and strength to the cell wall, enabling it to withstand external stresses and protect the plant cell from potential threats. The construction and modification of the cell wall are crucial in regulating plant responses to biotic stress. It has been reported that changes in the cell wall induced by pathogen invasion can trigger defense reactions, including the synthesis of antimicrobial compounds and the deposition of cell wall reinforcement materials [17]. Moreover, polysaccharides in the cell wall, such as pectin and low-methylesterified pectin, are involved in plant recognition and defense against pathogens [18]. Furthermore, the cell wall plays a vital role in resisting abiotic stress. In the previous reports, the thickness and composition of the cell wall can affect plant response to drought. Studies have shown that increasing the thickness and cellulose content of the cell wall can enhance the plant’s tolerance to drought [19,20].

Lignin, a complex phenolic polymer, is crucial for enhancing the rigidity of plant cell walls. Its synthesis is tightly regulated by a series of enzymes within the phenylpropane pathway, which includes phenylalanine ammonia lyase (PAL), cinnamate 4-hydroxylase (C4H), 4-hydroxycinnamate: CoA ligase (4CL), cinnamoyl-CoA reductase (CCR), cinnamyl alcohol dehydrogenase (CAD), and laccase (LAC) [21]. Plants often exhibit an increased lignin content in response to biotic and abiotic stress [22,23]. Studies have demonstrated that alterations in lignin content can impact plant resistance to pathogens. It has been shown that overexpression of *OsAAE3* in rice has been associated with reduced lignin content and increased susceptibility to rice blast [24]. In apples, MdMYB46 has been reported to enhance osmotic stress tolerance by promoting lignin deposition through the regulation of genes involved in lignin biosynthesis [25].

Pectin is a family of galacturonic acid-rich polysaccharides, including homogalacturonan, rhamnogalacturonan I, the substituted galacturonans rhamnogalacturonan II (RG-II), and xylogalacturonan (XGA). The biosynthesis of pectin is estimated to involve at least 67 transferase enzymes, including glycosyltransferases, methyltransferases, and acetyltransferases [26]. Due to its structure and cross-linking properties, pectin can influence cell wall hydration and porosity. Numerous studies have reported that plants respond to stress conditions by modifying the structure and content of pectin [27]. Ohara et al. (2021) [28] discovered that increased pectin content in rice leaves led to smaller intercellular gaps, acting as a defense mechanism against the rice blast fungus. Methylesterification levels determined by pectin methylesterase (PME) influence important cell wall properties related to plant tolerance to salt stress. In Arabidopsis, the knockout mutant of *PME31* exhibited lower transcript levels of several stress genes than the wild type under salt stress, suggesting that PME31 positively regulates salt stress tolerance [27].

Cellulose, a polysaccharide consisting of β-1,4-linked glucose chains, constitutes the cell wall’s most abundant and predominant biopolymer [29]. It is synthesized by the cellulose synthase complex (CSC), which is localized at the cytoplasmic membrane [30,31,32]. The cellulose skeleton serves as the functional core of the cell wall [32]. Research has revealed that overexpression of *OsCSLD4*, a gene encoding a cellulose synthase-like D4 protein in rice, enhances the expression of ABA synthesis genes and increases ABA levels, thereby improving rice’s salt tolerance [33]. Additionally, Kesten et al. (2017) [34] reported that microorganisms manipulate plant cellulose synthesis to weaken cell walls, facilitating their invasion. These insights imply a possible relationship between cellulose composition and a plant’s resilience to challenging conditions.

Hemicellulose, comprising approximately one-third of the cell wall biomass [35], primarily contains xyloglucan, xylan, mannan, glucomannan, and β-(1→3,1→4)-glucan. The biosynthesis of hemicellulose is facilitated by glycosyltransferases in the Golgi membrane [36]. Its primary biological function is reinforcing cell wall rigidity through interactions with cellulose and lignin. Studies have demonstrated that plants often increase their hemicellulose content within secondary cell walls in response to abiotic stress, thereby enhancing resistance [37]. Furthermore, Yang et al. (2021) [38] discovered that the pathogen *M. oryzae* secretes cell wall-degrading enzymes (CWDEs) to degrade hemicellulose in the cell wall. The degraded hemicellulose acts as damage-associated molecular patterns (DAMPs), triggering an immune response in the plants to defend against *M. oryzae* infection.

In conclusion, the composition of the rice cell wall alters in response to *M. oryzae* stress, either to facilitate fungal invasion or to trigger plant immune responses. We hypothesize that diploid and its autotetraploid rice may demonstrate different immune responses when stressed. Furthermore, the alterations in cell wall composition during salt stress may contribute to the plant’s adaptation to saline environments. Consequently, we expect that the cell wall composition of diploid and autotetraploid rice will be modified to adapt to salt stress conditions. Our study aims to (1) compare the phenotypic differences between diploid and autotetraploid rice; (2) assess the cell wall composition and related gene expression disparities between diploid and autotetraploid rice under normal conditions; (3) analyze the changes in major cell wall components and associated gene expression in response to *M. oryzae* and salt stress in both rice types. These investigations will enhance our understanding of the cell wall’s role in plant stress responses and shed light on the differential stress adaptation mechanisms between diploid and autotetraploid rice. Ultimately, these research findings will serve as crucial references and theoretical foundations for improving stress tolerance in rice.

## 2. Results

### 2.1. Alteration of Morphology and Polymer Content in Autotetraploid and Its Donor, Diploid Rice

**Figure 1:**
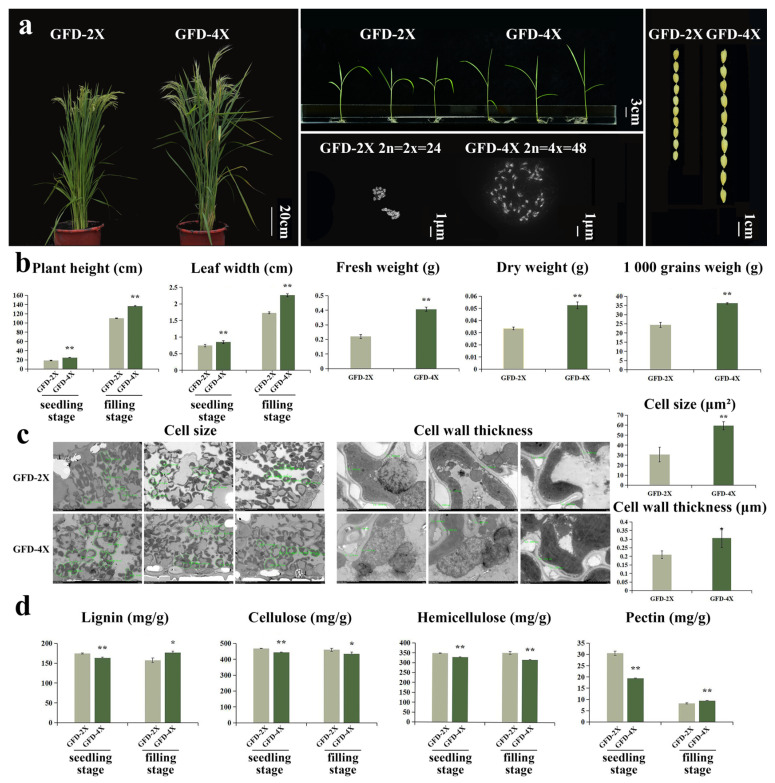
(**a**) GFD-2X and GFD-4X rice plants grown to the filling stage, bar = 20 cm; rice plants grown to the seedling stage, bar = 3 cm; chromosomes, bar = 1 μm; and seeds, bar = 1 cm. (**b**) GFD-2X and GFD-4X rice plant height at seedling and filling stages; leaf width at seedling and filling stages; seedling fresh weight; seedling dry weight; and the weight of a thousand seeds. Values are means ± SD (n = 5 biological replicates). (**c**) Cell size (bar = 10 μm) and cell wall thickness (bar = 2 μm) of GFD-2X and GFD-4X when grown to the filling stage. Values are means ± SD (n = 10 biological replicates). (**d**) Lignin, cellulose, hemicellulose, and pectin contents of GFD-2X and GFD-4X rice plants. Values are means ± SD (n = 3 biological replicates). * and ** indicate the level of significance of differences between GFD-2X and GFD-4X (*p* < 0.05 and *p* < 0.01) (Student’s *t* test).

In this study, the diploid japonica rice cultivar GFD-2X was naturally doubled to produce the tetraploid cultivar GFD-4X, which was stable planted in our laboratory. The morphological and polymer content was monitored in the GFD-4X cultivar. As depicted in Figure 1a,b, the results revealed phenotypic variation: GFD-4X had significantly larger seeds and a higher thousand-grain weight than GFD-2X. At the seedling stage, GFD-4X exhibited significantly greater plant height, broader leaves, and higher fresh and dry weights. At the filling stage, GFD-4X showed significantly wider leaves and greater plant height than GFD-2X.

To further investigate the morphological changes in autotetraploid rice, we performed transmission electron microscopy to observe the microscopic characteristics of leaves from both GFD-2X and GFD-4X cultivars (Figure 1c). At the filling stage of rice growth, the cell size and cell wall thickness of GFD-4X were significantly larger than those of GFD-2X. These findings provide compelling evidence suggesting that polyploid rice tends to cause cellular enlargement.

To investigate the disparities in cell wall composition between autotetraploid and its donor diploid rice, we analyzed the levels of critical components, including lignin, cellulose, hemicellulose, and pectin, at both the seedling and filling stages of rice growth (Figure 1d). At the seedling stage, we observed noteworthy variations in the cell wall composition between GFD-4X and GFD-2X. Specifically, the lignin content of GFD-4X was significantly lower, with a reduction of 6.63% compared to GFD-2X. Similarly, the cellulose content of GFD-4X exhibited a significant decrease of 5.43% compared to GFD-2X. Additionally, the hemicellulose content of GFD-4X was significantly lower, with a reduction of 5.64% compared to GFD-2X. Furthermore, the pectin content of GFD-4X was substantially lower, with a reduction of 36.70% compared to GFD-2X. Upon reaching the filling stage, we observed distinct differences in cell wall composition between GFD-4X and GFD-2X. Notably, the lignin content of GFD-4X was significantly higher, exhibiting an increase of 12.19% compared to GFD-2X. In contrast, the cellulose content of GFD-4X displayed a significant decrease of 5.73% compared to GFD-2X. Similarly, the hemicellulose content of GFD-4X exhibited a substantial decrease of 9.79% compared to GFD-2X. Interestingly, the pectin content of GFD-4X was significantly higher, with an increase of 14.41% compared to GFD-2X. Our findings provide compelling evidence for substantial alterations in the composition of the cell walls between diploid and autotetraploid rice, emphasizing the effects of polyploidization on cell wall structure and composition during different stages of rice growth.

### 2.2. Differential Expression Analysis of Genes Associated with Significant Cell Wall Components between Autotetraploid and Its Donor Diploid Rice

**Figure 2:**
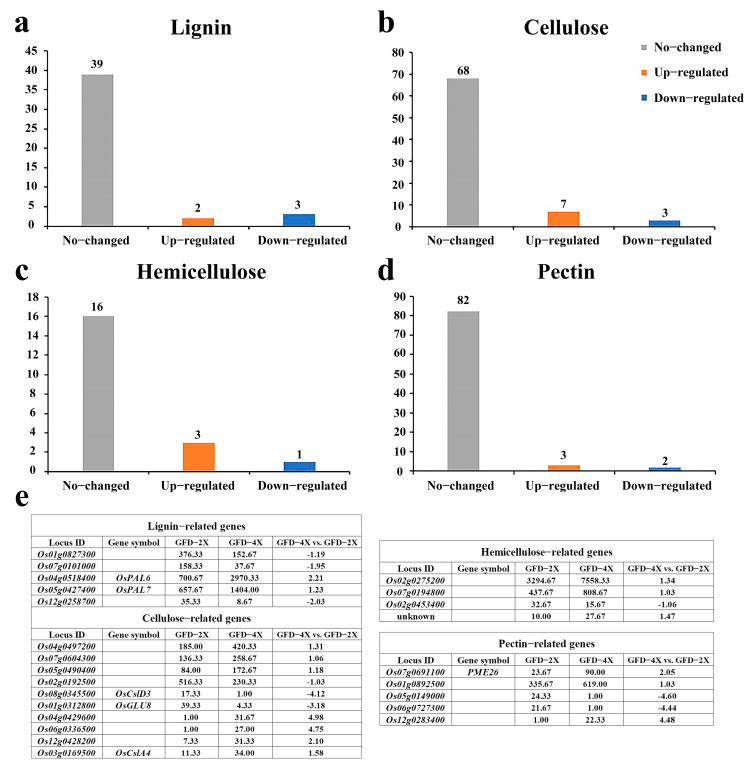
Differential expression analysis of genes related to cell wall major components between GFD-2X and GFD-4X. a-d represent a histogram of (**a**) lignin-related DEGs, (**b**) cellulose-related DEGs, (**c**) hemicellulose-related DEGs, and (**d**) pectin-related DEGs. (**e**) shows the expression levels of DEGs associated with significant components of the cell wall in GFD-2X and GFD-4X, and the differential multiples between GFD-4X and GFD-2X.

To further explore the disparities in cell wall composition between autotetraploid and its donor diploid rice, we conducted a comprehensive analysis of differentially expressed genes (DEGs) associated with the major components of the cell walls by transcriptome. The histograms were made based on the expression patterns of these genes in GFD-4X compared to GFD-2X, while the table was drawn to describe the expression levels and the difference multiples of these DEGs in both GFD-2X and GFD-4X (Figure 2).

In the comparison between GFD-4X and GFD-2X, five DEGs related to lignin were identified, with three genes showing down-regulation and two exhibiting up-regulation (Figure 2a,e). Furthermore, ten DEGs associated with cellulose were identified, among which three genes were down-regulated, and seven were up-regulated (Figure 2b,e). Four DEGs linked to hemicellulose were identified, with one gene showing down-regulation and three showing up-regulation (Figure 2c,e). Moreover, five DEGs associated with pectin were identified, with two genes down-regulated and three up-regulated (Figure 2d,e). These findings shed light on the differential expression patterns of genes involved in the significant components of the cell walls between diploid and tetraploid rice, providing valuable insights into the molecular mechanisms underlying cell wall composition variations in polyploid rice.

### 2.3. Phenotypic and Polymer Content Variation Occurred in Autotetraploid and Its Donor Diploid Rice under M. oryzae and Salt Stress Conditions

**Figure 3:**
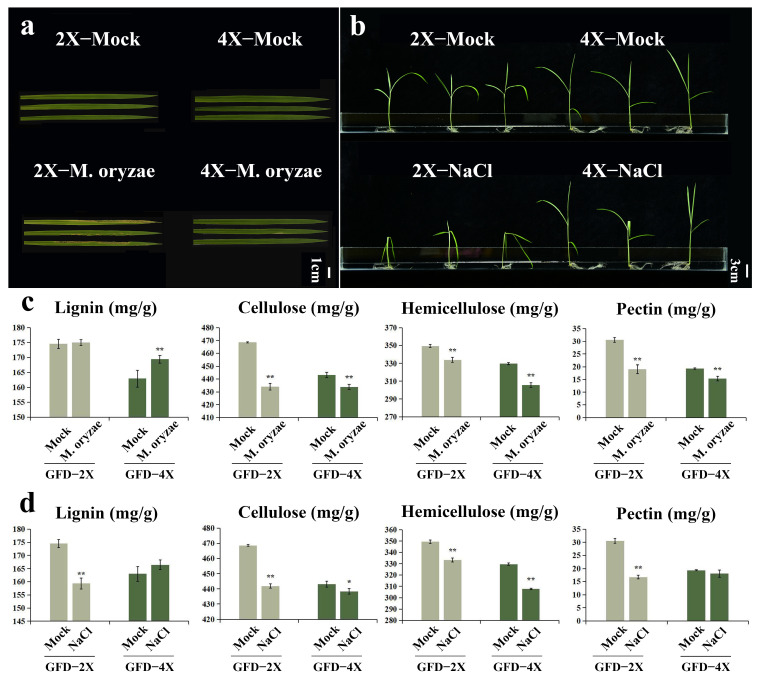
(**a**) Phenotypes of GFD-2X and GFD-4X rice leaves in typical habitats and under *M. oryzae* stress, bar = 1 cm. (**b**) GFD-2X and GFD-4X rice plant phenotypes in specific habitats and under salt stress, bar = 3 cm. (**c**) Changes in lignin, cellulose, hemicellulose, and pectin contents of GFD-2X and GFD-4X rice plants before and after *M. oryzae* stress. (**d**) Changes in lignin, cellulose, hemicellulose, and pectin contents of GFD-2X and GFD-4X rice plants before and after salt stress. Values are means ± SD (n = 3 biological replicates). * and ** indicate the level of significance of differences between treatment and control groups (*p* < 0.05 and *p* < 0.01) (Student’s *t* test).

In this study, we evaluated the response of diploid and its homologous tetraploid rice to *M. oryzae* stress and salt stress. 4X-Mock displayed wider leaves and taller plants than 2X-Mock (Figure 3a,b). 4X-*M. oryzae* showed fewer lesions and smaller damage areas than 2X-*M. oryzae* (Figure 3a). 4X-NaCl exhibited lower leaf wilting and a smaller stem tilt angle than 2X-NaCl (Figure 3b). These results suggest that tetraploid rice exhibits greater resistance to both *M. oryzae* stress and salt stress than diploid rice.

The contents of the major components of the cell walls of GFD-2X and GFD-4X were quantified in response to the induction of *M. oryzae* stress or salt stress. Under *M. oryzae* stress induction, the lignin content exhibited no significant change in GFD-2X. In comparison, it showed a highly significant increase of 3.94% in GFD-4X. The cellulose content showed a significant decrease of 7.38% in GFD-2X and 2.07% in GFD-4X. Similarly, the hemicellulose content exhibited a substantial decrease of 4.45% in GFD-2X and 7.33% in GFD-4X. Furthermore, the pectin content showed a significant decrease of 37.70% in GFD-2X and 20.94% in GFD-4X (Figure 3c).

Under salt stress induction, the lignin content showed a significant decrease of 8.72% in GFD-2X, while it exhibited no substantial change in GFD-4X. The cellulose content showed a significant decrease of 5.69% in GFD-2X and 1.05% in GFD-4X. Similarly, the hemicellulose content showed a significant decrease of 4.61% in GFD-2X and 6.61% in GFD-4X. Additionally, the pectin content displayed a substantial decrease of 45.35% in GFD-2X, while it showed no significant change in GFD-4X (Figure 3d).

### 2.4. Expression of Genes Associated with Significant Cell Wall Components in Autotetraploid and Its Donor Diploid Rice under M. oryzae and Salt Stress Conditions

**Figure 4:**
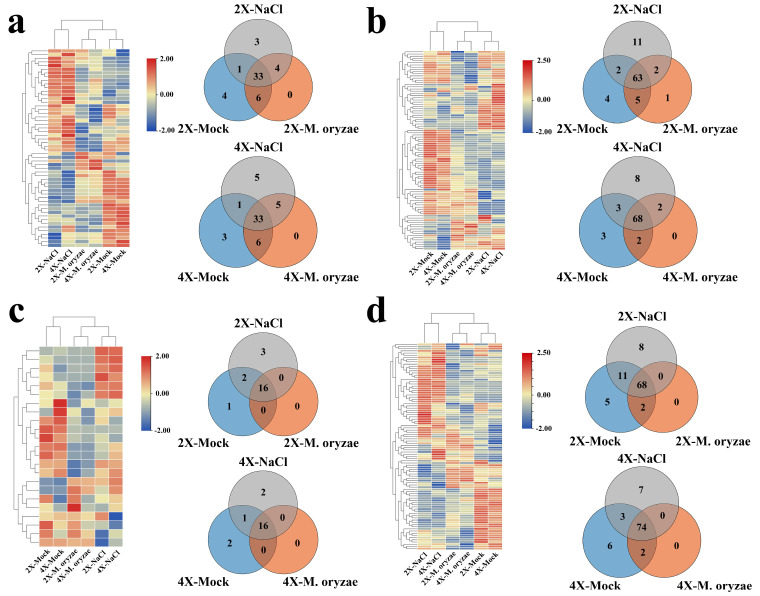
The expression of genes related to major components of the cell wall of GFD-2X and GFD-4X under normal growth conditions, *M*. *oryzae* stress condition, and salt stress condition. Different treatment conditions of GFD-2X and GFD-4X (**a**) lignin-associated, (**b**) cellulose-associated, (**c**) hemicellulose-associated, and (**d**) pectin-associated expressed genes in Venn diagrams and heat maps of gene expression levels.

Venn diagrams were made using a count max threshold of >30 for gene expression to identify the genes related to the major cell wall components expressed in GFD-2X and GFD-4X under different treatment conditions. Heatmaps were generated to exhibit the expression levels of these genes (Figure 4). Comparative analysis revealed that the expression patterns of GFD-2X and GFD-4X were similar under the same treatment condition.

There were 37 lignin-related genes, 65 cellulose-related genes, 16 hemicellulose-related genes, and 68 pectin-related genes showing the same expression pattern in 2X-*M. oryzae* and 2X-NaCl. For GFD-4X, 38 lignin-related genes, 70 cellulose-related genes, 16 hemicellulose-related genes, and 74 pectin-related genes showed the same expression pattern in 4X-*M. oryzae* and 4X-NaCl. These findings indicate that more genes related to the significant components of the cell wall were induced by *M. oryzae* stress and salt stress in GFD-4X than GFD-2X.

**Figure 5:**
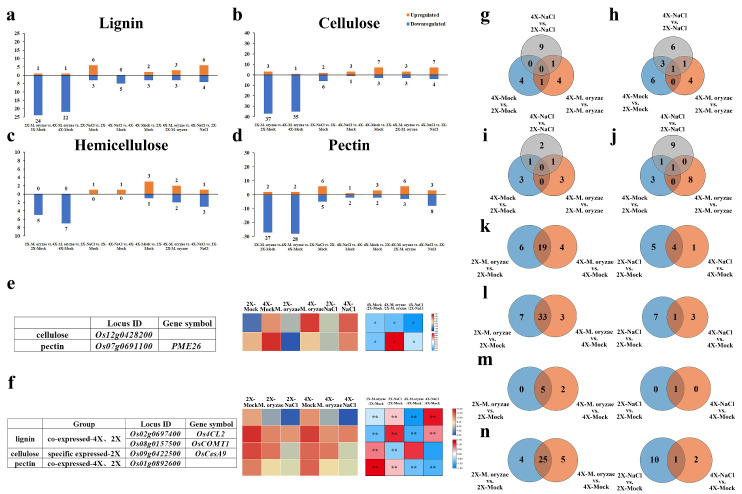
Comparison and analysis of DEGs related to cell wall components of GFD-2X and GFD-4X under normal habitat, *M. oryzae* stress, or salt stress. a-d are histograms of DEGs associated with (**a**) lignin, (**b**) cellulose, (**c**) hemicellulose, and (**d**) pectin, respectively, with horizontal coordinates for two-by-two comparisons of the different species and vertical coordinates for the number of DEGs, where orange columns denote up-regulated genes and blue columns denote down-regulated genes. (**e**) Heatmap of DEGs related to cell wall major components between diploids and tetraploids. (**f**) Heatmap of DEGs related to major cell wall components induced by both *M. oryzae* stress and salt stress. g-j represent Venn diagrams of (**g**) lignin-, (**h**) cellulose-, (**i**) hemicellulose-, and (**j**) pectin-associated DEGs between GFD-2X and GFD-4X under different treatment conditions, respectively. k-n are Venn diagrams of (**k**) lignin-, (**l**) cellulose-, (**m**) hemicellulose-, and (**n**) pectin-associated DEGs between treatment and control groups in GFD-2X and GFD-4X under *M. oryzae* stress (**left**) or salt stress (**right**), respectively. * and ** indicate the level of significance of differences (*p* < 0.05 and *p* < 0.01) (Student’s *t* test).

To investigate the differential expression of lignin-, cellulose-, hemicellulose-, and pectin-related genes of GFD-2X and GFD-4X under different stress conditions, as well as between GFD-2X and GFD-4X under the same stress condition, two-by-two comparative histogram were generated based on the number of DEGs and up- and down-regulation (Figure 5a–d). The results showed that in 2X-*M. oryzae* vs. 2X-Mock, there were 25 lignin-related DEGs (1 up-regulated), 40 cellulose-related DEGs (3 up-regulated), five hemicellulose-related DEGs (no up-regulated genes), and 29 pectin-related DEGs (2 up-regulated). Similarly, in 4X-*M. oryzae* vs. 4X-Mock, there were 23 lignin-related DEGs (1 up-regulated), 36 cellulose-related DEGs (1 up-regulated), seven hemicellulose-related DEGs (no up-regulated genes), and 30 pectin-related DEGs (2 up-regulated). In 2X-NaCl vs. 2X-Mock, there were nine lignin-related DEGs (6 up-regulated), eight cellulose-related DEGs (2 up-regulated), one hemicellulose-related DEG (1 up-regulated), and 11 pectin-related DEGs (6 up-regulated). In 4X-NaCl vs. 4X-Mock, there were five lignin-related DEGs (no up-regulated genes), four cellulose-related DEGs (3 up-regulated), one hemicellulose-related DEG (1 up-regulated), and three pectin-related DEGs (1 up-regulated). In 4X-*M. oryzae* vs. 2X-*M. oryzae*, there were six lignin-related DEGs (3 up-regulated), six cellulose-related DEGs (3 up-regulated), four hemicellulose-related DEGs (2 up-regulated), and nine pectin-related DEGs (6 up-regulated). Finally, in 4X-NaCl vs. 2X-NaCl, there were ten lignin-related DEGs (6 up-regulated), 11 cellulose-related DEGs (7 up-regulated), four hemicellulose-related DEGs (1 up-regulated), and 11 pectin-related DEGs (3 up-regulated).

Venn diagrams were constructed to compare the DEGs associated with the major components of the cell wall between GFD-2X and GFD-4X under various treatment conditions (Figure 5g–j). The analysis revealed that two genes, *Os12g0428200* (related to cellulose) and *Os07g0691100* (related to pectin), exhibited differential expression patterns between GFD-2X and GFD-4X under different treatment conditions. Notably, the expression level of these genes in GFD-4X was consistently higher than that of GFD-2X (Figure 5e).

DEGs associated with the major components of the cell wall in GFD-2X and GFD-4X under *M. oryzae* or salt stress were compared and analyzed (Figure 5k–n). When induced by *M. oryzae* stress, a total of 19 lignin-related genes, 33 cellulose-related genes, five hemicellulose-related genes, and 25 pectin-related genes were found to be differentially expressed in both GFD-2X and GFD-4X. Additionally, six lignin-related genes, seven cellulose-related genes, and four pectin-related genes were explicitly differentially expressed in GFD-2X. In comparison, four lignin-related genes, three cellulose-related genes, two hemicellulose-related genes, and five pectin-related genes were explicitly differentially expressed in GFD-4X. Under salt stress induction, four lignin-related genes, one cellulose-related gene, one hemicellulose-related gene, and one pectin-related gene were differentially expressed in GFD-2X and GFD-4X. Moreover, five lignin-related genes 7, seven cellulose-related genes, and ten pectin-related genes were explicitly differentially expressed in GFD-2X. For the GFD-4X, one lignin-related gene, three cellulose-related genes, and two pectin-related genes were explicitly differentially expressed.

Venn diagrams (Supplementary Figure S1) were generated to illustrate the specific and common DEGs associated with the major components of the cell wall in GFD-2X and GFD-4X under *M. oryzae* stress and salt stress conditions (Figure 5k–n). Moreover, a heatmap was constructed to exhibit the expression levels of the identified DEGs in response to *M. oryzae* stress and salt stress, considering the fold change difference between the treatment and control groups (Figure 5f). The analysis revealed that specific genes, such as the diploid-specific cellulose-related gene *Os09g0422500*, as well as the diploid- and tetraploid-common lignin-related genes *Os08g0157500* and *Os02g0697400*, and pectin-related gene *Os01g0892600*, were down-regulated in response to *M. oryzae* stress and salt stress.

### 2.5. Correlation Analysis of Cell Wall Main Component Content and Expression Levels of Related Genes

**Figure 6:**
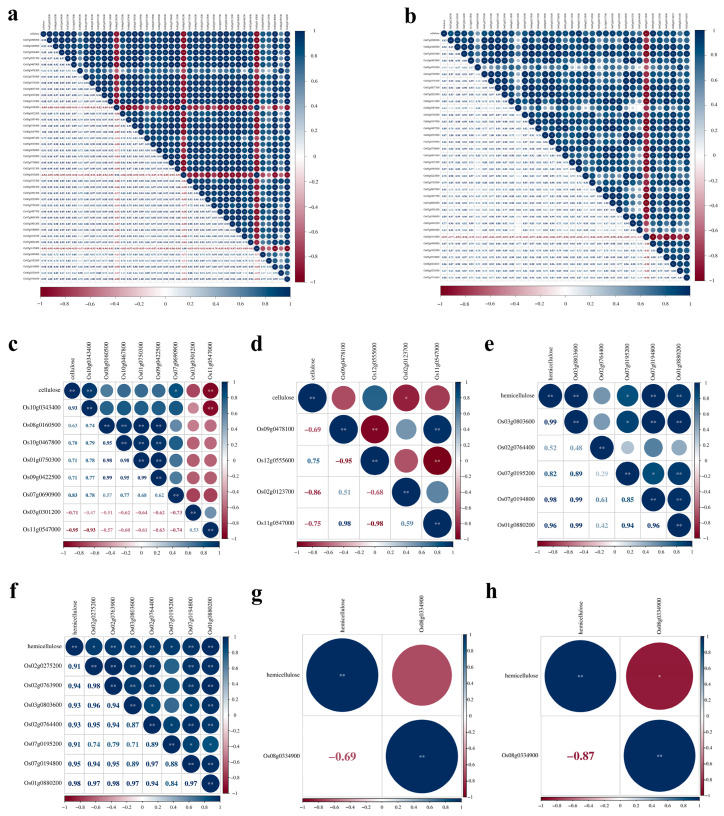
Correlation analysis of cellulose and hemicellulose content and expression levels of related genes. a-d respectively denoted in (**a**) 2X-*M. oryzae* vs. 2X-Mock, (**b**) 4X-*M. oryzae* vs. 4X-Mock, (**c**) 2X-NaCl vs. 2X-Mock, and (**d**) 4X-NaCl vs. 4X-Mock, correlation analysis of cellulose content and expression levels of related genes. e-h respectively denoted in (**e**) 2X-*M. oryzae* vs. 2X-Mock, (**f**) 4X-*M. oryzae* vs. 4X-Mock, (**g**) 2X-NaCl vs. 2X-Mock, (**h**) 4X-NaCl vs. 4X-Mock, correlation analysis of hemicellulose content and expression levels of related genes. * and ** indicate significant levels of association (*p* < 0.05 and *p* < 0.01).

A correlation analysis was performed to explore the relationship between the content of cell wall main components and the expression levels of related genes under *M. oryzae* stress or salt stress. The content of cellulose, hemicellulose, lignin, and pectin in the cell walls of GFD-2X and GFD-4X under different treatment conditions was quantified. The expression levels of genes associated with these cell wall components were also determined. The correlation coefficients (r) and significance test results of cellulose, hemicellulose, lignin, and pectin contents with the expression levels of the related genes are shown in Figure 6 and Supplementary Figure S2. In 2X-*M. oryzae* vs. 2X-Mock, 33 genes, including *Os07g0208500*, exhibited a significant positive correlation with cellulose content, while three genes, such as *Os06g0336500*, showed a significant negative correlation with cellulose content (Figure 6a). Additionally, four genes, including *Os03g0803600*, displayed a significant positive correlation with hemicellulose content (Figure 6e). Moreover, 24 genes, such as *Os06g0193200*, exhibited a significant positive correlation with pectin content, while two genes, such as *Os12g0466200*, showed a significant negative correlation with pectin content (Supplementary Figure S2c).

In the 4X-*M. oryzae* vs. 4X-Mock comparison, 27 genes, such as *Os07g0208500*, displayed a significant positive correlation with cellulose content, whereas *Os05g0115800* exhibited a significant negative correlation with cellulose content (Figure 6b). Furthermore, seven genes, such as *Os02g0275200*, displayed a significant positive correlation with hemicellulose content (Figure 6f). Additionally, seven genes, including *Os04g0416900*, showed a significant negative correlation with lignin content (Supplementary Figure S2b). Moreover, 20 genes, such as *Os07g0691100,* exhibited a significant positive correlation with pectin content, while two genes, such as *Os11g0153100*, showed a significant negative correlation with pectin content (Supplementary Figure S2d).

In comparing 2X-NaCl vs. 2X-Mock, two genes, *Os10g0343400* and *Os11g0547000*, exhibited significant positive and negative correlations with cellulose content (Figure 6c). Similarly, three genes, including *Os08g0157500* and *Os12g0259800*, were significantly, positively, and negatively correlated with lignin content, respectively (Supplementary Figure S2a). Additionally, two genes, such as *Os01g0892600*, displayed a significant positive correlation with pectin content, while four genes, such as *Os01g0788400*, were significantly and negatively correlated with pectin content (Supplementary Figure S2e).

In comparing 4X-NaCl vs. 4X-Mock, *Os02g0123700* was significantly and negatively correlated with cellulose content, indicating its potential role in regulating cellulose synthesis under salt stress (Figure 6d). Moreover, *Os08g0334900* exhibited a negative correlation with hemicellulose content, suggesting its involvement in hemicellulose metabolism under salt stress conditions (Figure 6h).

To further determine the accuracy of RNA-seq analysis of the two rice varieties under different treatment conditions, we randomly selected seven genes for qRT-PCR amplification: *Os01g0639200*, *Os09g0400000*, *Os10g0578200*, *Os01g0312500*, *Os01g0788400*, *Os02g0626100*, and *Os02g0738900*. The results revealed that the expression levels of the seven selected genes were consistent with the results of RNA-seq determination (Supplementary Figure S3).

## 3. Discussion

Plant polyploidy is one of the essential driving forces in plant evolution and has significant implications for the survival and adaptation of both wild and cultivated plants [39]. Polyploidy can increase plant size and growth rate and enhance their ability to adapt to environmental stresses [40,41]. Therefore, studying polyploid plants’ characteristics and adaptive differences is essential for understanding plant evolution and improvement.

This study compared the differences in several phenotypic traits between diploid and autotetraploid rice plants, including plant height, leaf width, cell size, and cell wall thickness. Additionally, we examined their adaptive response to stress from *M. oryzae* stress and salinity. Consistent with previous research findings, we found that GFD-4X rice plants exhibited larger organs and more vital adaptability (Figure 1 and Figure 3). Furthermore, we conducted a comprehensive analysis of the contents of the major cell wall components and the expression of related genes in GFD-2X and GFD-4X, both before and after treatments, and compared these between the diploid and autotetraploid rice. We explained the reasons for the differential performance of diploid and autotetraploid rice under stress conditions from the perspective of the cell wall. The cell wall is an essential component of plant cells, providing structural support and participating in plant responses and adaptations to the external environment. Therefore, changes in the cell wall may be a key factor leading to the differential performance of diploid and tetraploid rice in terms of adaptability and stress resistance.

Further research has demonstrated that lignin and cellulose are the primary components of plant cell walls, playing a crucial role in their structure and function. Lower lignin content can lead to loosening the cell wall structure, increasing its plasticity and extensibility, enabling plants to better adapt to changes in the external environment. Additionally, the content and arrangement of cellulose determine the rigidity and strength of the cell wall. Lower cellulose content may increase the fragility of the cell wall, but it also enhances its plasticity, allowing plants to withstand adverse conditions better. Serapiglia et al. (2014) [42] found that triploid and tetraploid willows have lower lignin content than diploid willows. Plant mutants with impaired cellulose synthesis typically exhibit enhanced resistance to stress [43]. This could be attributed to triggering a series of cellular signaling pathways by impairing cellulose synthesis and activating plant defense mechanisms. These defense mechanisms include increased production of antioxidants, activation of stress-related genes, and regulation of plant hormone synthesis and signaling [43]. In our study, the more vital adaptability of GFD-4X rice under stress conditions may be associated with its impaired cellulose synthesis. Furthermore, we observed that GFD-4X rice has lower lignin and cellulose content and exhibits more vital adaptability under stress conditions. This suggests that changes in cell wall composition may be an essential factor contributing to the differential performance of diploid and tetraploid rice in terms of adaptability and stress resistance. Further investigation into the composition and function of the cell wall is essential for understanding the mechanisms underlying plant adaptability and stress resistance, providing valuable insights for plant breeding and improvement.

Numerous studies have demonstrated that plant cell walls exhibit high dynamism and are not inert entities, playing a crucial role in plant responses to biotic and abiotic stresses [44]. Furthermore, the composition and structure of plant cell walls can be modulated in response to various stimuli [45]. In this study, we observed alterations in the content of major cell wall components and the expression of associated genes following *M. oryzae* or salt stress induction. Notably, these changes exhibited significant differences between diploid and homologous tetraploid plants. Li et al. illustrated that rice plants enhance their resistance against *M. oryzae* by accumulating lignin, thereby increasing cell wall thickness [46]. Consistent with these findings, our study revealed a similar trend in lignin content for the disease-resistant GFD-4X variety before and after *M. oryzae* stress. At the same time, no significant difference was observed in the disease-sensitive GFD-2X variety (Figure 3c). Plants have evolved a cell wall integrity (CWI) maintenance system to monitor cell wall integrity, facilitating their adaptation to adverse environmental conditions without compromising cell wall organization. Salt stress represents an abiotic stressor that can severely impair CWI, and the plant’s ability to sustain CWI is critical for salt tolerance [47]. Our study found no significant difference in lignin and pectin content between GFD-4X before and after salt stress. In contrast, GFD-2X exhibited a substantial reduction in lignin and pectin content. This suggests that GFD-4X possesses a superior capacity to maintain an average level of lignin and pectin content under salt stress, potentially contributing to its enhanced salt tolerance compared to GFD-2X. Yang et al. demonstrated that *M. oryzae* infects host plants by secreting cell wall degradation enzymes (CWDEs) that target cellulose and hemicellulose in the cell wall, triggering the release of damage-associated molecular patterns (DAMPs). These DAMPs function as “danger” signals, which can be perceived by CWI-maintaining pattern recognition receptors (PRRs) localized on the cell surface, activating the plant’s immune response and inducing the expression of defense genes [38]. Consistent with previous studies, both GFD-2X and GFD-4X exhibited a significant decrease in cellulose and hemicellulose contents under the *M. oryzae* stress.

Under normal growth conditions, *M. oryzae* stress conditions, and salt stress conditions, the expression of genes associated with significant cell wall components differed between GFD-2X and GFD-4X. Analysis of the correlation between the content of major cell wall components and gene expression in GFD-2X and GFD-4X revealed that the DEG *Os11g0547000*, which was up-regulated by salt stress in both diploids and tetraploids, was enriched in the BP category “negative regulation of cellulose biosynthetic process” (GO: 2001007) (Supplementary Table S1). This gene showed a highly significant negative correlation with cellulose content in GFD-2X, while there was no significant correlation with cellulose content in GFD-4X (Figure 6c,d). This may be one of the reasons why the decrease in cellulose content was lower in GFD-4X than in GFD-2X. The DEG *Os08g0334900*, which was up-regulated by salt stress in both diploids and tetraploids, was enriched in the BP categories “xyloglucan biosynthetic process” (GO:0009969) and “cell wall organization” (GO:0071555) (Supplementary Table S1). The gene showed no significant correlation with hemicellulose content in GFD-2X. Still, it showed a significant negative correlation in GFD-4X (Figure 6g,h), suggesting that this may be one of the reasons for the higher rate of decrease in hemicellulose content in GFD-4X than GFD-2X. Furthermore, we found that *M. oryzae* stress-induced differential expression of more genes involved in regulating major cell wall component content than salt stress (Figure 6, Supplementary Figure S2).

The pectin methylesterase (PME) gene *Os07g0691100* exhibited differential expression between GFD-2X and GFD-4X under various treatment conditions (Figure 5e,j). Specifically, this gene was up-regulated in GFD-4X compared to GFD-2X under both *M. oryzae* stress and salt stress. PMEs have been extensively studied for their involvement in plant responses to biotic and abiotic stresses [27]. Coculo et al. (2023) [48] proposed that increased PME activity leads to pectin demethylation, resulting in cell wall reinforcement and damage signaling, thereby enhancing plant resistance to pathogens. Furthermore, demethylation of pectin by PMEs generates negatively charged carboxyl groups that can bind to Na+ ions in salt solution, effectively preventing Na+ influx into the cytoplasm and enhancing plant resistance to salt stress [49,50]. Based on these findings, it is hypothesized that the up-regulation of *Os07g0691100* in GFD-4X may modify the pectin structure to confer stress resistance. In contrast, the cellulose synthase catalytic subunit gene *Os09g0422500* was specifically down-regulated in GFD-2X under both *M. oryzae* stress and salt stress induction (Figure 5f). Previous studies have demonstrated that the knockdown of this gene affects cellulose synthesis in the secondary cell wall of rice [51,52]. This reduction in cellulose content observed in GFD-2X under stress conditions could be attributed to the down-regulation of *Os09g0422500*. Notably, this decrease in cellulose content was more pronounced in GFD-2X compared to GFD-4X.

Interestingly, under salt stress treatment, the cinnamyl alcohol dehydrogenase (CAD) gene *Os09g0399800*, a DEG familiar to both GFD-2X and GFD-4X, was up-regulated in GFD-2X but down-regulated in GFD-4X. The specific function of this gene has not been investigated in rice, but CAD is known to catalyze monomeric lignin biosynthesis [53]. Previous studies have demonstrated that apple enhances tolerance to osmotic stress by promoting lignin deposition by regulating lignin biosynthesis-related genes [25]. However, it should be noted that the previous studies have primarily focused on diploids, and tetraploids, such as GFD-4X, may have different regulatory mechanisms due to genome doubling. Further experiments are required to explore this hypothesis. The gene *Os12g0258700* encodes the laccase precursor protein and was expressed only in GFD-2X in average conditions. However, under *M. oryzae* stress, both GFD-2X and GFD-4X showed induced expression, with GFD-4X exhibiting higher expression levels than GFD-2X. Laccase encoded by this gene reportedly involved rice plants’ catabolism of herbicide residues [54]. However, there is currently no literature linking this gene to disease resistance. The strong induction of *Os12g0258700* expression in GFD-4X under *M. oryzae* stress observed in our study is of research significance. Further experiments are necessary to investigate the potential role of this gene in plant disease resistance.

Our study found that the changes in cell wall composition and related gene expression under biotic stress were more closely related than those under abiotic stress. When pathogens invade plant cells, a series of defense responses are triggered, including the remodeling and reinforcement of the cell wall. These defense responses can lead to changes in cell wall composition and gene expression related to cell wall synthesis and remodeling. In contrast, abiotic stress, such as salt stress, is usually caused by environmental factors. Salt stress may lead to thinning and damage to the cell wall. Still, this change is more related to the physical properties of the cell wall, such as strength and plasticity, rather than directly related to changes in cell wall composition and gene expression. Therefore, from the perspective of the cell wall, changes in cell wall composition and related gene expression under biotic stress are more significant and related. This suggests that remodeling and reinforcing the cell wall may be an essential strategy for cultivating crops resistant to biotic stress. Further research on the effects of biotic stress on cell wall composition and related gene expression can help us better understand the plant’s response mechanisms to biotic stress and provide theoretical support for cultivating crops with stronger resistance.

## 4. Materials and Methods

### 4.1. Plant Material and Stress Treatments

In this study, diploid (GFD-2X) and tetraploid rice (GFD-4X) were used. GFD-4X was a natural mutant autotetraploid rice derived from GFD-2X screened in the field and self-pollinated for six generations. Root-tip cells were utilized for karyotypic analysis, and chromosomes were stained with 4,6-diamidino-2-phenylindole (DAPI) staining as previously described [55]. Two hundred fully developed seeds were selected and germinated on moist germination paper for 2–3 days. Subsequently, the germinated seedlings were transferred to a half-strength Hoagland nutrient solution to saturate for 20 days and grown under controlled environmental conditions to reach the three-leaf stage, with a photoperiod of 16 h light and 8 h darkness at a temperature of 28 ± 1 °C. In brief, rice seeds were sowed in a plastic container with a hydroponic culture apparatus (60–70% humidity). For salt stress treatment, fifty GFD-2X and GFD-4X plants each were treated with 125 mM NaCl for 24 h, and samples were collected [14]. For the rice blast stress treatment, another fifty strains of GFD-2X and GFD-4X each were inoculated with a spore suspension of 1 × 10^5^ spores/mL, prepared from the rice blast strain “20180211” obtained from the College of Plant Protection, Jilin Agricultural University. Samples were collected at 36 h post-inoculation [56]. All samples are immediately frozen in liquid nitrogen and stored at −80 °C until use.

### 4.2. Determination of Agronomic Traits

For GFD-2X and GFD-4X, 2000 whole seeds were selected to measure thousand-seed weight. Plant height, leaf width, fresh weight, and dry weight were determined at the seedling stage of rice growth. To investigate additional agronomic traits, 30 plants of GFD-2X and GFD-4X were cultivated in the field, and plant height and leaf width measurements were taken at the rice growth filling stage. Each of the measurements mentioned above was performed in five biological replicates.

### 4.3. Determination of Lignin, Cellulose, Hemicellulose, and Pectin Content

The content of cell wall main components was determined in seedling rice plants under normal growth conditions, *M. oryzae* stress conditions, and salt stress conditions, as well as in rice plants grown naturally in the field until the filling stage. The fresh samples were subjected to thermal treatment at 105 °C for 10–15 min, followed by drying at 80 °C until a constant weight was achieved. The dried samples were then crushed and sieved. Subsequently, the samples were treated with ethanol, NaOH, ice acetic acid, and hydroxylamine hydrochloride, then centrifuged at 5000 rpm for 5 min. The resulting supernatant was diluted 10-fold with ice acetic acid, and the lignin content was determined using an enzyme marker at 280 nm.

The samples were treated with 80% ethanol, pure acetone, dimethyl sulfoxide, and distilled water. The resulting supernatant was discarded, and sulfuric acid was added to the residue. After 30 min of standing, the supernatant was collected. Anthrone was added and mixed with the samples, which were then subjected to a water bath at 95 °C for 10 min. Subsequently, the samples were cooled to room temperature, and the cellulose content was determined using an enzyme marker at 620 nm.

Calcium nitrate was added to the sample, followed by a water bath at 90 °C for 10 min. The mixture was then centrifuged, and the supernatant was discarded. Distilled water was added to the residue and vortexed for 2 min. The mixture was centrifuged at 25 °C, 8000× *g* for 5 min, and the supernatant was discarded. This step was repeated. The resulting residue was dried at 80 °C and then treated with hydrochloric acid. A water bath at 90 °C was performed for 1 h, then natural cooling. NaOH solution was added, and the mixture was centrifuged to obtain the supernatant. Phenol, sodium bisulfite, potassium sodium tartrate, NaOH, 3,5-dinitro salicylic acid solution, and distilled water were thoroughly mixed with the supernatant. The mixture was subjected to a water bath at 90 °C for 5 min, followed by natural cooling and centrifugation. The hemicellulose content in the supernatant was determined using an enzyme marker at 540 nm.

The samples were treated with a 95% ethanol solution and heated in an oven at 95 °C for 30 min. After cooling to room temperature, the mixture was centrifuged at 8000 rpm for 5 min, and the supernatant was discarded. The step was repeated. The residue was then treated with distilled water and subjected to a water bath at 50 °C for 30 min. After cooling, the mixture was centrifuged at 8000 rpm for 10 min, and the supernatant was collected to determine soluble pectin content. 1mL of extraction solution was added and thoroughly mixed with the remaining residue. The mixture was then subjected to a water bath at 95 °C for 1 h. After cooling, the mixture was centrifuged at 8000 rpm for 10 min, and the supernatant was collected to determine the original pectin content.

The sample supernatant containing soluble and original pectin was treated with concentrated sulfuric acid, mixed thoroughly, and subjected to a water bath at 90 °C for 10 min. After cooling, carbazole was added, and the mixture was allowed to stand at 25 °C for 30 min. Distilled water was added, and the content of soluble pectin and original pectin was determined using an enzyme marker at 530 nm. The sum of the two values represented the total pectin content. The abovementioned measurements were conducted using a BioTek Epoch 2 enzyme labeling instrument (BioTek Instruments, Winooski, VT, USA), with three biological replicates performed for each sample.

### 4.4. Transmission Electron Microscopy Observations

The samples used for transmission electron microscopy (TEM) observation were GFD-2X and GFD-4X rice leaves grown to seedling and filling stages, which were grown under normal habitat conditions. Leaf samples of 1 mm^3^ were manually cut and immediately fixed in an electron microscope fixative at four °C for 2–4 h. After fixation, the samples were rinsed three times for 15 min each in 0.1 M phosphate buffer saline (PBS) with a pH of 7.4. Subsequently, the samples were fixed in 1% osmium acid solution in 0.1 M PBS at room temperature (20 °C) for 2 h, then three rinsed in 0.1 M PBS for 15 min each. The tissues were then dehydrated sequentially in 50%, 70%, 80%, 90%, 95%, and 100%-ethanol solutions for 15 min each. A mixture of acetone and 812 embedding agents in a 1:1 ratio was prepared for overnight infiltration, followed by overnight infiltration in pure 812 embedding agents. The samples were then embedded and polymerized at 60 °C for 48 h. Ultrathin 60–80 nm sections were cut using a microtome and double stained with uranium-lead (2% uranyl acetate saturated aqueous solution and lead citrate) for 15 min each. The sections were air-dried overnight at room temperature and subsequently observed under a transmission electron microscope. Images were collected and analyzed using Image-Pro Plus 6.0 software (Media Cybernetics, Inc., Rockville, MD, USA). Cell area (μm²), and cell wall thickness (μm) was measured on a 6k two μm scale. Each measurement was performed with ten biological replicates.

### 4.5. RNA Extraction and Sequencing Analysis

Total RNA isolation was performed on GFD-2X and GFD-4X rice plants under normal growth conditions (0 h), salt stress condition (treated with 24 h), and *M. oryzae* stress condition (treated for 36 h). A total of six samples were subjected to transcriptomic analysis and referred to as 2X-Mock, 4X-Mock, 2X-NaCl, 4X-NaCl, and 2X-*M. oryzae*, and 2X-*M. oryzae*, respectively.

Total RNA was extracted with the TRIzol reagent following the manufacturer’s protocol (Life Technologies Invitrogen, Carlsbad, CA, USA). The purity, concentration, and integrity of the RNA samples were assessed using NanoDrop 2000 and Agilent 2100 instruments. Only high-quality RNA samples were used for cDNA library construction. After library qualification, PE150 mode sequencing was performed using the Illumina NovaSeq6000 sequencing platform. The sequencing data were filtered to obtain clean data, and the high-quality reads were aligned to the Oryza_sativa.MSU_v7.0.genome.fa using HISAT2 software [57]. Differentially expressed genes (DEGs) were identified using the criteria of |log2 (fold_change)| > 1 and *p* < 0.05. Functional classification and enrichment analysis of DEGs associated with major cell wall components by Gene Ontology (GO) were conducted to analyze their biological processes, cellular composition, and molecular functions (Supplementary Table S1). A total of 18 libraries, consisting of three biological replicates for each of the six sample types, were included in the analysis.

### 4.6. Statistical Analysis

In this study, statistical data analysis, such as organ size and cell wall major component content, was performed using Student’s *t* test in the DPS data processing system [58]. Heatmaps and Venn diagrams based on the gene expression of each sample were generated using TBtools software (v1.120) [59]. The content of the main components of the cell wall and the expression of related genes were statistically analyzed using bioinformatics (http://www.bioinformatics.com.cn, accessed on 10 May 2023). Pearson’s correlation was used to estimate the association between them. The statistical significance threshold was set at *p* < 0.05.

### 4.7. qRT-PCR Validation

To validate the RNA sequencing data, a quantitative real-time polymerase chain reaction (qRT-PCR) was performed on seven randomly selected cell wall significant component-related genes using the SYBR Green I PCR master mix kit (TaKaRa, Tokyo, Japan) according to a previously reported method and repeated three times [60]. Gene-specific primer pairs were downloaded from qPrimerDB (Supplementary Table S2). The results of the qRT-PCR analysis are shown in Supplementary Figure S3.

## 5. Conclusions

In this study, we found that the organs of the autotetraploid rice were significantly larger than those of diploid rice. This indicates that polyploidy has a significant promoting effect on plant organ development. Meanwhile, we also found that the content of the main components of the cell wall (lignin, cellulose, hemicellulose, and pectin) in autotetraploid rice was significantly lower than in diploid rice. This may be due to the differences in the regulation of cell wall synthesis and degradation mechanisms in autotetraploid rice. Through transcriptome analysis, we found differentially expressed genes related to the main components of the cell wall in diploid and autotetraploid rice. The differential expression of these genes may lead to differences in cell wall composition. To further compare changes in cell wall composition between diploid and autotetraploid rice under stress conditions, we analyzed differences in cell wall composition and related gene expression under *M. oryzae* and salt stress. The results showed that, compared to diploid rice, the cell wall composition of autotetraploid rice changed more significantly under *M. oryzae* and salt stress. This further confirms the advantage of autotetraploid rice in responding to biotic stress. Our study also found that autotetraploid rice had lower lignin and cellulose content, and exhibited more vital adaptability under stress conditions. This may be due to changes in cell wall composition leading to differences in adaptability and stress resistance in autotetraploid rice. In conclusion, further research on the composition and function of the cell wall is of great significance for understanding plants’ adaptability and stress resistance mechanisms. These research findings provide valuable information for plant breeding and improvement, helping cultivate crop varieties more adapted to their environment and more stress-tolerant.

## Data Availability

The datasets generated and analyzed in this study are available at PRJNA1026748 (https://www.ncbi.nlm.nih.gov/bioproject/PRJNA1026748, accessed on 11 October 2023) and PRJNA1010456 (https://www.ncbi.nlm.nih.gov/bioproject/PRJNA1010456, accessed on 29 August 2023).

## Supplementary Materials

The following supporting information can be downloaded at: https://www.mdpi.com/article/10.3390/plants12233976/s1, Figure S1: Venn diagrams of differentially expressed genes related to major cell wall components that are specific to GFD-2X (left), specific to GFD-4X (middle), and common to GFD-2X and GFD-4X (right) under the induction of *M. oryzae* stress and salt stress conditions, a-d represents (a) lignin, (b) cellulose, (c) hemicellulose and (d) pectin related genes, respectively; Figure S2: Correlation analysis of lignin and pectin content and expression levels of related genes. a-b respectively denoted in (a) 2X-NaCl vs. 2X-Mock, (b) 4X-*M. oryzae* vs. 4X-Mock, correlation analysis of lignin content and expression levels of related genes. c-e respectively denoted in (c) 2X-*M. oryzae* vs. 2X-Mock, (d) 4X-*M. oryzae* vs. 4X-Mock, (e) 2X-NaCl vs. 2X-Mock, correlation analysis of pectin content and expression levels of related genes. * and ** indicate significant levels of association (*p* < 0.05 and *p* < 0.01); Figure S3: (a) The results of seven genes expression amplified by qRT-PCR. * and ** indicate the level of significance of differences between treatment and control groups (*p* < 0.05 and *p* < 0.01) (Student’s *t* test). (b) Comparison of RNA-seq results and qRT-PCR analysis of gene expression levels; Table S1: Results of GO enrichment of differentially expressed genes related to lignin, cellulose, hemicellulose and pectin in GFD-2X and GFD-4X induced by rice blast fungus stress or salt stress; Table S2: Primers used for qRT-PCR assays.
