# Supplementary material for: A Comparative Analysis of Major Cell Wall Components and Associated Gene Expression in Autotetraploid and Its Donor Diploid Rice (Oryza sativa L.) under Blast and Salt Stress Conditions
Source: Plants (Basel). 2023 Nov 26;12(23):3976. doi: 10.3390/plants12233976 (PMC10708163; doi:10.3390/plants12233976)
Supplement: Supplementary file 1 [file plants-12-03976-s001.zip › Illustrations.pdf]

Supplementary Table S1. Results of GO enrichment of differentially expressed genes related to lignin, cellulose, hemicellulose and pectin in GFD-2X and GFD-4X induced by rice blast fungus stress or salt stress.

Supplementary Table S2. Primers used for qRT-PCR assays.

Supplementary Figure S1. Venn diagrams of differentially expressed genes related to major cell wall components that are specific to GFD-2X (left), specific to GFD-4X (middle), and common to GFD-2X and GFD-4X (right) under the induction of *M. oryzae* stress and salt stress conditions, a-d represents (a) lignin, (b) cellulose, (c) hemicellulose and (d) pectin related genes, respectively.

Supplementary Figure S2. Correlation analysis of lignin and pectin content and expression levels of related genes. a-b respectively denoted in (a) 2X-NaCl vs. 2X-Mock, (b) 4X-*M. oryzae* vs. 4X-Mock, correlation analysis of lignin content and expression levels of related genes. c-e respectively denoted in (c) 2X-*M. oryzae* vs. 2X-Mock, (d) 4X-*M. oryzae* vs. 4X-Mock, (e) 2X-NaCl vs. 2X-Mock, correlation analysis of pectin content and expression levels of related genes. * and ** indicate significant levels of association ($p<0.05$ and $p<0.01$).

Supplementary Figure S3. (a) The results of seven genes expression amplified by qRT-PCR. * and ** indicate the level of significance of differences between treatment and control groups ($p < 0.05$ and $p < 0.01$) (Student's *t* test). (b) Comparison of RNA-seq results and qRT-PCR analysis of gene expression levels.
