# Supplementary figures and images for: A Comparative Analysis of Major Cell Wall Components and Associated Gene Expression in Autotetraploid and Its Donor Diploid Rice (Oryza sativa L.) under Blast and Salt Stress Conditions
Source: Plants (Basel). 2023 Nov 26;12(23):3976. doi: 10.3390/plants12233976 (PMC10708163; doi:10.3390/plants12233976)

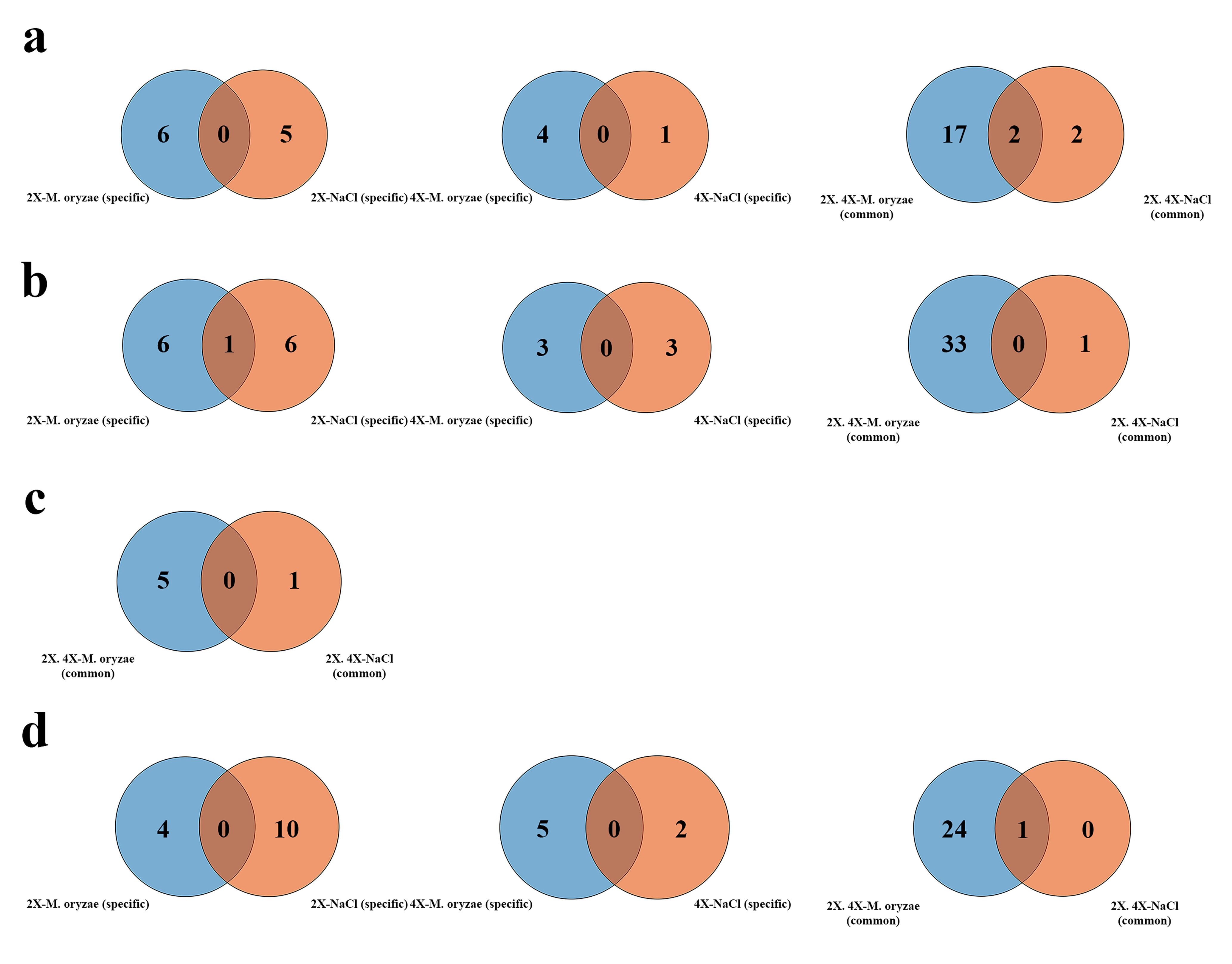

Supplement: Supplementary file 1 [file plants-12-03976-s001.zip › Supplementary Figure S1.tif]

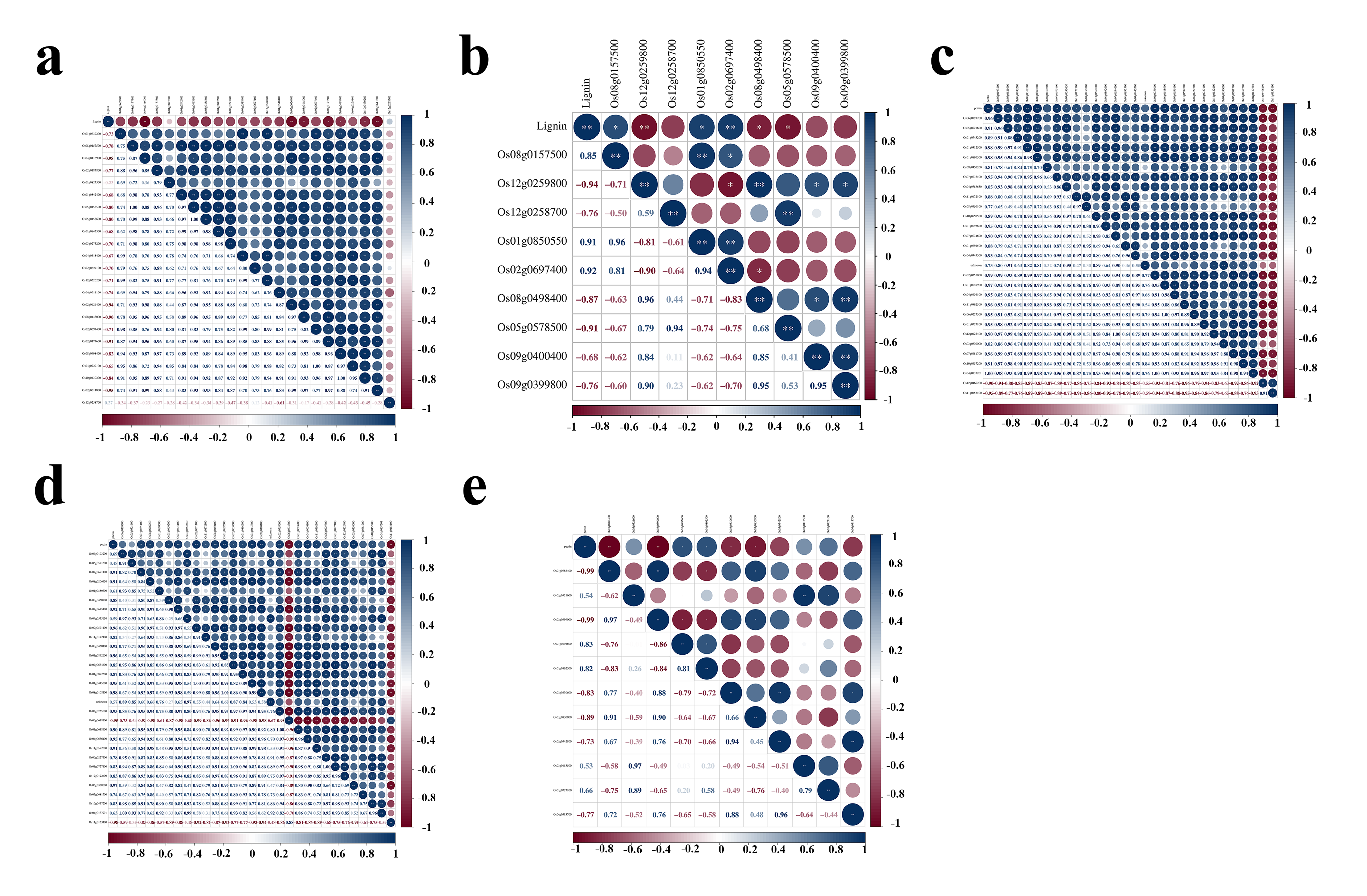

Supplement: Supplementary file 1 [file plants-12-03976-s001.zip › Supplementary Figure S2.tif]

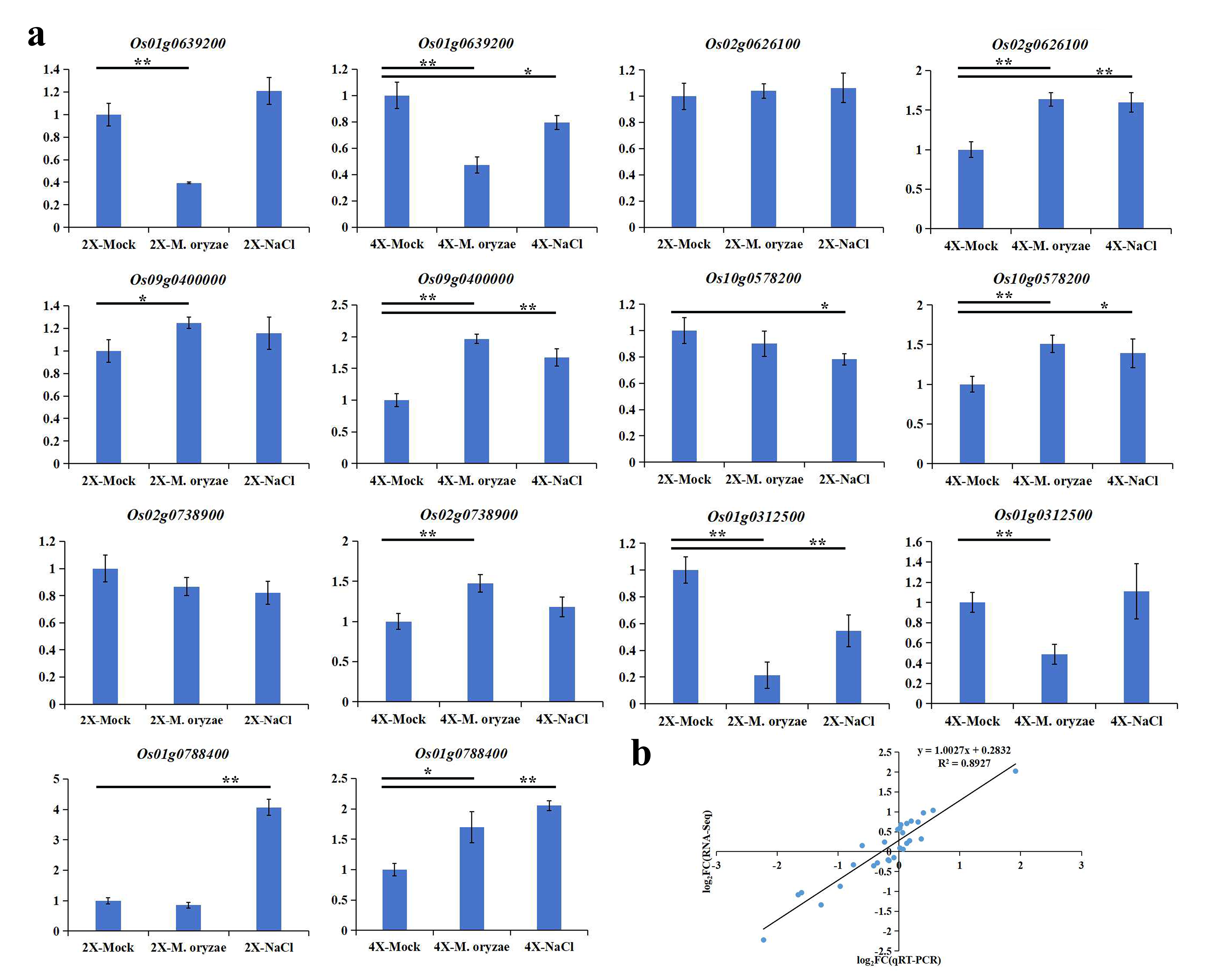

Supplement: Supplementary file 1 [file plants-12-03976-s001.zip › Supplementary Figure S3.tif]
